# Extracellular DNA (eDNA). A Major Ubiquitous Element of the Bacterial Biofilm Architecture

**DOI:** 10.3390/ijms22169100

**Published:** 2021-08-23

**Authors:** Davide Campoccia, Lucio Montanaro, Carla Renata Arciola

**Affiliations:** 1 Laboratorio di Patologia delle Infezioni Associate all’Impianto, IRCCS Istituto Ortopedico Rizzoli, via di Barbiano 1/10, 40136 Bologna, Italy; lucio.montanaro@unibo.it; 2 Department of Experimental, Diagnostic and Specialty Medicine, University of Bologna, via San Giacomo 14, 40126 Bologna, Italy

**Keywords:** extracellular DNA, eDNA, biofilm matrix, extracellular polymeric substance, EPS, DNA-binding proteins, orthopedic implant infections

## Abstract

After the first ancient studies on microbial *slime* (the name by which the biofilm matrix was initially indicated), multitudes of studies on the morphology, composition and physiology of biofilms have arisen. The emergence of the role that biofilms play in the pathogenesis of recalcitrant and persistent clinical infections, such as periprosthetic orthopedic infections, has reinforced scientific interest. Extracellular DNA (eDNA) is a recently uncovered component that is proving to be almost omnipresent in the extracellular polymeric substance (EPS) of biofilm. This macromolecule is eliciting unprecedented consideration for the critical impact on the pathogenesis of chronic clinical infections. After a systematic review of the literature, an updated description of eDNA in biofilms is presented, with a special focus on the latest findings regarding its fundamental structural role and the contribution it makes to the complex architecture of bacterial biofilms through interactions with a variety of other molecular components of the biofilm matrix.

## 1. Introduction

Bacterial biofilm-associated infections are known to be particularly difficult to treat with conventional antibiotics and represent a major clinical challenge. This type of infection is primarily known to occur in the presence of implant biomaterials, mainly used in the orthopedic clinical field, and other indwelling devices, where solid material surfaces constitute a substratum for bacterial anchorage, colonization and subsequent establishment of bacterial communities encased within protective extracellular polymeric matrices [1,2,3]. However, similar biofilms, where bacteria form aggregates enveloped in polymeric substances, can be observed even in infections that develop in the absence of foreign bodies as in the case of osteomyelitis, cystic fibrosis, periodontitis, rhinosinusitis, and infective endocarditis [4,5]. This second circumstance also occurs in pathologic conditions such as septic arthritis, when biofilms form in joint synovial fluids by suspended bacteria [6]. In the absence of any supporting abiotic surface, biofilms are mobile and develop in form of flocs. Under both circumstances, i.e., in the presence as well as in the absence of a solid substratum, intercellular adhesion factors such as adhesins and the extracellular polymeric substance (EPS) play a fundamental role in mediating bacterial cells aggregation, taking part to the composition of the biofilm matrix architecture and contributing to its many protective functions. In clinical biofilms, EPS often comprises not only polymers produced by bacteria, but also components of the extracellular matrix (ECM) of host tissues or molecules released from host cells such as leukocytes. Bacteria have special ability in recycling these basic molecules and repurposing them for their own scopes. Once occupied a niche in human tissues, they take advantage of the many substances available in the host interstitial milieu to gain protection. These substances are largely represented by organic molecules that constitute the ECM architecture, remnant materials generated from tissue damage and from the death of host cells, substances actively secreted by bacteria or residual components derived from bacterial cell lysis processes.

Biofilm organic components include different categories of substances, among them: polysaccharides [7,8], proteins [9,10], nucleic acids [11,12,13], teichoic acids [14], and even lipids [15,16]. Biofilm formation is influenced by numerous factors, among them: the strain’s genetic background and the presence of genes that are mobile among lineages [17,18,19], a different constitutive expression of the genes [19], the presence or the absence of the insertion element IS256 within the intercellular adhesion (*ica*) locus [20], the presence of mobile genetic elements [21], the production of lytic enzymes [22], the modulation by quorum sensing systems [23,24,25,26,27], these often presenting many allelic variants, and cues from the external environment [28,29,30]. A remarkable diversity characterizes biofilm EPS in different bacterial species, but even strain types of the same species as well as the same bacterial strain under varying environmental conditions have been found capable to form different biofilms. These alternative forms of biofilm, indicated by some authors as “biofilm morphotypes” [18], may vary in structure and EPS composition and offer flexibility for environmental adaptation.

There is a high biodiversity in the EPS that takes part to the molecular structure of the biofilm matrix, with distinct exopolysaccharides and proteic factors being expressed in different Gram-positive and Gram-negative bacterial species. On the contrary to other known EPS components, extracellular DNA (eDNA) has emerged to be a nearly universal component of microbial biofilms across environmental and pathogenic species, these including not only bacteria but also fungi (e.g., *Candida albicans* [31]). Initially thought a remnant of lysed cells retained in biofilms, eDNA was more recently discovered to take part to supramolecular structures such as stable filamentous networks [32]. Surprisingly, the different taxa of microorganisms have independently redirected and repurposed the use of DNA, sometimes reaching convergent evolutive solutions/functions, but frequently completely reinventing the ways this molecule is released in the outer space and it interacts with the other EPS components. Due to a high degree of complexity, despite all the intense research work conducted over several decades, in many species of microorganisms the mechanisms of release and regulation of eDNA still remain unknown [33].

A profound comprehension of bacterial extracellular matrix architecture represents the first critical step to the development of new effective strategies for preventing and treating persistent chronic infections. In this regard, eDNA and its interactions with eDNA-binding molecules are becoming primary targets. In clinical infections, eDNA is not only a common trait of the extracellular matrix of biofilms established on biomaterials surfaces, but also a main component of most biofilms growing in suspension. Concentrations of up to 4 mg/mL of eDNA have been reported in the lungs of patients affected by cystic fibrosis, nearly an order of magnitude greater than in normal tissues [34]. This polyanionic molecule plays an important role in facilitating early adhesion to abiotic material surfaces, enabling the attachment of bacterial cells even to less adhesive hydrophobic surfaces and thus supporting the initial, but also fundamental, steps of surface colonization. It promotes intercellular aggregation and plays a fundamental structural function for the integrity of the biofilm architecture.

eDNA is an extremely versatile molecule that represents much more than a mere inert structural element of the biofilm architecture. As a real multitask player, eDNA has been found to mediate horizontal gene transfer [35,36], bind and sequester cations, including magnesium, from the surrounding environment [37], neutralize important cationic effector molecules of the innate immunity such as antimicrobial peptides (AMPs) [38,39], actively contribute to biofilm tolerance by restricting the diffusion of antimicrobials [40,41,42], acidify the local environment and promote antibiotic resistant phenotypes [43], interact with cells of the host immune system and condition both the innate and the cell-mediate immune response [44,45,46,47,48,49,50], function as a precursor of leukocidal molecules [51], guide biofilm spreading [52], and serve as a nutrient source during starvation [53], promote efficient extracellular electron transfer by phenazines [54]. The interest on eDNA is further enhanced by some recent findings that enlighten an existing link between its interaction with amyloid proteins and the pathogenesis of autoimmune disorders [55,56].

Over the years, the general concept of biofilm architecture has impressively evolved. In the early overly simplified view, biofilm was initially conceived as clumps of sessile microorganisms encased in an amorphous accumulation of viscous sticky or gummy substances also referred to as slime for the muddy consistency [57,58]. The “slime layer present around the organisms” was progressively associated to bacterial virulence [59,60], while parallel studies were running to investigate slime chemical composition in clinically relevant species [61]. The adoption of the term *film* to indicate a layer of sessile bacterial microorganisms attached onto a trickling filter substrate, comprising a self-secreted matrix, is generally attributed to Mack et al. and dates back to 1975 [62]. The role of biofilm formation in the pathogenesis of infections associated to implant related materials became new object of study [63,64] and so became the morphology and ultrastructure of biofilms formed on biomaterial surfaces in clinical infections [65]. With the contribution of emerging techniques such as confocal laser scanning microscopy (CLSM), the direct observation of the morphology of biofilms formed by different bacteria under static or dynamic conditions became feasible, unveiling the heterogeneous structure varying with time and space of bacterial sessile communities and their basic building block, the microcolony. With this important impulse, the development of bacterial biofilms and their physiology started to be better understood and new definitions and concepts were formulated [66,67,68]. In 2002, Whitchurch et al. [12] observed that eDNA is required for the initial establishment of *Pseudomonas aeruginosa* biofilm and suggested that also other biofilms could specifically release DNA. Nonetheless, the extracellular biofilm matrix was still seen as a mere accumulation of hydrated and differently distributed EPS and not as the assembly of a well-defined molecular architecture, where polymeric molecules develop specific interactions and bind to each other based on their affinity and specific physico-chemical characteristics, conferring to the biofilm stability and resistance to mechanical stress. Only recently, with a more detailed knowledge of the many components that take part to the composition of the biofilm matrix and their possibilities of reciprocal molecular interactions, a new type of exploration has started and eDNA has progressively emerged as a principal structural element of biofilm architecture. After ten years, this review article follows our previous paper [69] focused on extracellular DNA in biofilms and intends to provide a more updated and detailed overview on the mechanisms that bacteria adopt to release/secrete DNA in the outer space, repurposing this versatile molecule to architect their stable and highly protective biofilms.

In view of the ubiquity of this important EPS component, eDNA can be regarded as an ideal broad-spectrum molecular target for preventing and treating biofilm-based infections [70]. Indeed, eDNA represents a critical molecule for the structural integrity of biofilm and its shielding effects, which confer tolerance to physical and chemical stress as well as protection from host immune response and medical therapies.

## 2. Criteria Adopted for the Review of the Literature

**Figure 1:**
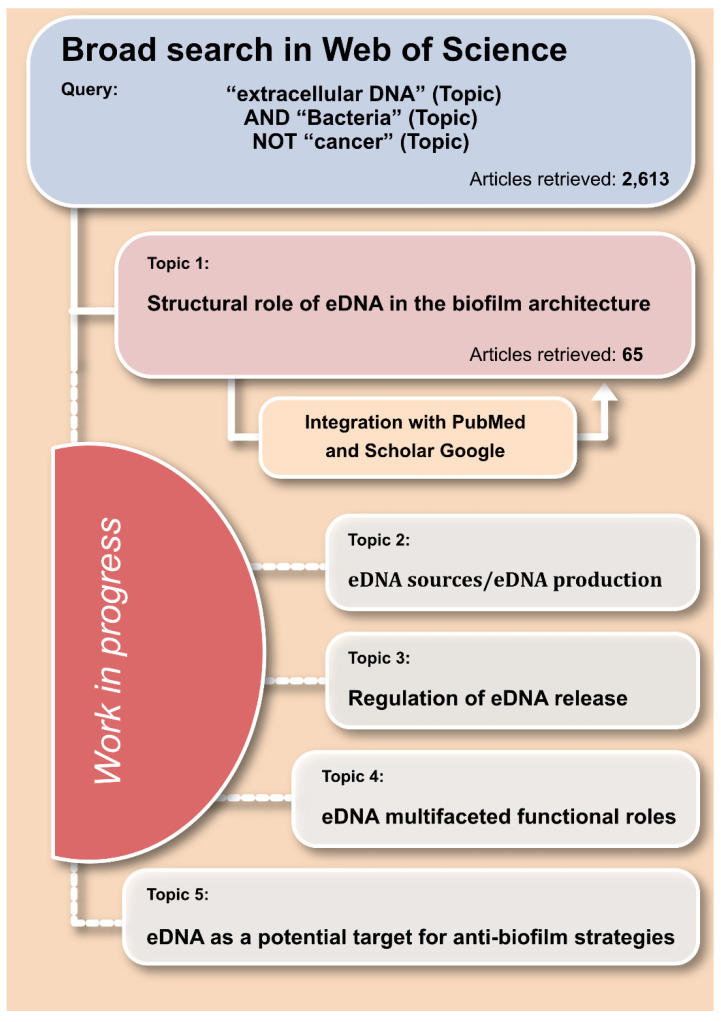
Scheme of the systematic review.

This review was generated in a context of a broader systematic survey aimed at gathering all the information currently available in the literature on extracellular DNA, with special reference to the physiology and functions in bacterial biofilms. The bibliographic research was primarily conducted by Web of Science and then extended by PubMed and Google Scholar. The initial broad query adopted in Web of Science was as follows: “extracellular DNA” (Topic) AND “Bacteria” (Topic) NOT “cancer” (Topic) (Figure 1). An alert was set, and the search results were regularly updated until the 5 May 2021. Overall, 2613 article entries were obtained from this broad research. Experimental articles were thoroughly screened based on the title and the abstract. The content of review articles was examined, assessing, and eventually including their relevant citations. All pertinent manuscripts were clustered together based on the relevant treated topics into the following categories: “eDNA sources/eDNA production”, “regulation of eDNA release”, “structural role of eDNA in the biofilm architecture”, “eDNA multifaceted functional roles”, and a further special category concerning “eDNA as a potential target for anti-biofilm strategies”. The present systematic review was based on the articles selected as relevant to the category “structural role of eDNA in the biofilm architecture”.

## 3. eDNA Structural Role in Biofilm Architecture

For diverse bacterial species, the main EPS components of biofilm extracellular matrix have progressively been identified, with eDNA appearing ubiquitous. Consequently, current research efforts are progressively being focused on establishing if and how each single polymeric substance structurally integrates with the others to originate the typical biofilm architectural frame. In this regard, eDNA has clearly emerged to play a pivotal role and develop different physico-chemical interactions not only with main EPS components of the biofilm extracellular matrix, but also with factors expressed on the bacterial surfaces and with abiotic surfaces. Thus, among other important roles, eDNA contributes to bacterial aggregation promoting intercellular adhesion [71], mediates attachment to abiotic material surfaces enabling bacterial anchorage [72] and stabilizes the biofilm architecture [73,74,75] by interlacing different polymeric components. Hu et al. [76] studied *Myxococcus xanthus* biofilms and reported as the direct interactions of eDNA with exopolysaccharides enhance the physical strength and the resistance of biofilms to biological stress. Further work by Peterson et al. [77] has highlighted a distinguishable role of different matrix constituents in stress relaxation. Under mechanical stress, eDNA would appear to modulate its interactions with other EPS components and, in this way, control its contribution to viscoelastic relaxation. Therefore, in view of recent advancements in the comprehension of the organization of the biofilm, eDNA would not simply accumulate in the extracellular space as an inert polymer with filtering and sheltering functions. Rather, it would form molecular complexes and interact with a multiplicity of chemical substances, generating a sort of crosslinked auto-setting gel with heterogeneous features, undergoing progressive modifications, and remodeling during different phases of biofilm maturation.

Gradients of pH generated by bacteria growing in hypoxic conditions would influence electrostatic charges of eDNA and determine its local propensity to interact with other cationic substances. The formation of gels consisting of polyelectrolyte complexes starting from charged biopolymers is well established and has been exploited for the fabrication of biocompatible hydrogels for biomedical applications [78,79]. A long molecule (if fully stretched out, the bacterial chromosome would measure nearly 1000 times the size of a bacterial cell [80]), eDNA may generate a sort of main grid and attract and help in retaining soluble and diffusible polycationic molecules otherwise lost to the biofilm matrix construction. The particular importance of a polyanionic molecule such as eDNA in the biofilm architecture is suggested by heparin, another polyanionic molecule, which was found to mimic eDNA in binding to cell-surface *Staphylococcus aureus* proteins and enhance biofilm formation [81]. However, in bacterial biofilm matrices the contribution to the architecture stability is provided by additional, more complex physico-chemical interactions involving different categories of compounds, which are described in the following dedicated paragraphs. The interactions of eDNA with biofilm exopolysaccharides and proteins will be treated in the following paragraphs.

## 4. Interaction of eDNA with Biofilm Exopolysaccharides

Given its polyanionic nature, some molecular interactions developed by eDNA with other biofilm components are mediated by its electrostatic charge. In staphylococcal species such as *S. aureus* and *Staphylococcus epidermidis*, the polysaccharide intercellular adhesin (PIA) represents a main exopolysaccharide biofilm component. PIA, whose expression is associated to the *ica*ADBC locus [25], consists of poly-β-(1,6)-*N*-acetylglucosamine, a polysaccharide that, during its processing, is partially de-acetylated by the enzyme IcaB and acquires a net positive charge. In *S. epidermidis* biofilms, PIA is recognized to be a main EPS component of the extracellular matrix. Conversely, different past investigations supported the idea that *S. aureus* would produce biofilms through two main distinct pathways, alternatively leading to the assembly of a polysaccharide-based biofilm morphotype or an eDNA/protein biofilm morphotype [82,83]. The polysaccharide-based biofilm morphotype was reported as predominantly observed in methicillin-sensitive *S. aureus* (MSSA) strains, while the eDNA/protein biofilm morphotype would be more typical of methicillin-resistant *S. aureus* strains (MRSA). However, very recent data by Mlynek et al. [84] would suggest that these forms of biofilm extracellular matrices are not mutually exclusive. Rather, the relative proportions of matrix components and sensitivity to extracellular-matrix-degrading agents such as DNase I and proteinase are governed by the conditions in which biofilms develop and the differential expression of factors associated to biofilm formation or their respective degradation/catabolism (e.g., proteases). Interestingly, Mlynek and colleagues propose a model where, in reason of their respective electrostatic charge, PIA directly interacts with eDNA. In turn, eDNA binds one or more lipoproteins in the biofilm [85]. This electrostatic net model would finally support and combine some earlier observations suggesting the occurrence of both eDNA/protein [86] and eDNA/PIA complexation [17]. In 2018, the work of Sugimoto et al. [70] earlier highlighted a broad impact of eDNA on the biofilm of clinical MSSA and MRSA strains. They reported even in PIA-dependent biofilms less but sufficient levels of eDNA to promote biofilm formation and maintain biofilm structures. Levels were found to depend on the strain and on the culture condition and not always correlated with the extent of biofilm biomass. Conversely, in a past investigation assessing the composition of biofilms formed by 55 clinical *S. epidermidis* strains, levels of eDNA and PIA were found to some degree correlated [17]. This is somehow consistent with the findings in *S. aureus* of Mlynek and colleagues [84], suggesting a synergistic function in biofilm formation and bacterial aggregation by eDNA and PIA, under the control of the global regulator CodY. Very recently, Skovdal et al. [87] have questioned the role played by PIA and other *S. epidermidis* adhesins such as Embp under in vivo real clinical conditions with respect to in vitro laboratory conditions. The authors showed as, in an *S. epidermidis* 1585 WT strain, which was deficient in *ica*ADBC, and in its derivative strain lacking the *embp* gene, host factors in human plasma could restore bacterial aggregation and vancomycin tolerance. Biofilms formed in human plasma were reported to be loosely attached and consisting of suspended aggregates. It should be said that, traditionally, loosely attached bacterial aggregates that deposit during sedimentation under static conditions are normally removed by gentle rinsing before the assessment of the firmly adhered biofilm on a material surface.

Thus, the appeal of Flemming et al. [88] to reconsider and revise the definition of biofilm in all its possible manifestations is overly due. Moreover, bacterial flexibility to adapt to different environmental conditions often relies on redundant mechanisms to aggregate, condition the external environment, and generate their protected niches. Opportunistic pathogens such as staphylococci, *Escherichia coli* and *P. aeruginosa* can inhabit different environmental niches, including drinking water distribution systems [89] and natural waters [90]. It is conceivable that depending on the local availability of polymeric substances, bacteria may rely on their own production of EPS or on available external sources. It is certainly true that there is need to gain a more complete information on the composition of bacterial biofilms in vivo in clinical samples and that in vitro models should more closely simulate the in vivo human physiological fluids found at the different anatomic sites [91].

Important molecular interactions between eDNA and biofilm exopolysaccharides have been described for the biofilms of other important pathogens. In *P. aeruginosa* biofilms, depending on the strain type, the architecture of the extracellular matrix is variously characterized by the presence of different exopolysaccharides, these including: alginate, a polyanionic polysaccharide consisting of guluronic and mannuronic acids and typically overproduced in mucoid strains; Psl polysaccharide (the product of the polysaccharide synthesis locus, *Psl*), a neutral mannose-rich polysaccharide; and pellicle (Pel), a polycationic, partially de-*N*-acetylated polysaccharide consisting of *N*-acetylglucosamine and *N*-acetylgalactosamine [92,93,94,95].

During the morphogenesis of biofilm matrix architecture, thick-structured biofilms are formed even by mutant strains producing just one or two of the above-mentioned polysaccharides. The sole exception is observed when the mutant is uniquely capable of producing alginate [96]. Psl exopolysaccharide typically accumulates at the periphery of the 3D-structured mushroom-like microcolonies that form during biofilm maturation [97], where it appears to accomplish a scaffolding structural function. Conversely, the microcolony center remains Pls-free and occupied by bacterial cells. Cell death and lysis occurring in this central area of the microcolony result in an abundant release of eDNA. Interestingly, Jennings et al. [93] found that the polycationic Pel exopolysaccharide develops ionic interactions with eDNA as earlier described for PIA in staphylococci, determining the formation of molecular cross-links. The authors observed that Pel and eDNA generally colocalize to the stalk of the microcolony structure. Pel distribution is largely influenced by the strain type and its respective ability of expressing Psl. In fact, a mutant strain defective for the *psl* gene expressed substantial amounts of Pel, which could be detected even at the periphery of the biofilm, this documenting as Pel can eventually compensate and replace Psl functions. Jennings and colleagues [93] could not ascertain differences in Pel transcriptional control that could explain its prevalent distribution in microcolonies stalk. They offered the alternative explanation that, at least in part, pH gradients with their influence on ionic polymer charge and presence of eDNA drive the Pel/eDNA interactions, generating the structural core of the stalk. In this regard, Reichhardt & Parsek [94] demonstrated as Pel becomes positively charged only with a pH at or below the isoelectric point (corresponding to 6.3), which can be reached at the core of the microcolony. Through the interaction with eDNA, Pel appears to contribute to the formation of meshwork-like structures and influence biofilm cell density and/or the compactness of the biofilm [96]. Wang et al. [98] reported that even Psl can interact with eDNA and, in in vitro grown *P. aeruginosa* biofilms, form fiber-like structures, where the two polymers colocalize. Psl-based fibers were further confirmed in vivo and found to emanate from the body of the biofilms [99]. However, the nature of the interaction between the Psl and eDNA remains unexplained. In contrast, Reichhardt and Parsek [94] found that exogenous DNA did not accumulate in negative control biofilms formed by a PAO1Δ*wspF* Δ*pel* pBAD*psl* mutant strain, which lacks Pel and overexpresses Psl, this suggesting that only Pel could specifically interact with eDNA. Better established are the interactions of both Psl [94] and Pel [95] with the extracellular adhesin CdrA, implicated in promoting biofilm formation through biofilm matrix cross-linking of different exopolysaccharides. Dispersion of *P. aeruginosa* cells in the late biofilm maturation stage is finally achieved through the degradation of both Pel and Psl polysaccharides [100].

An important recent discovery was that Pel-DNA aggregates, which form at pH 6.3 by salmon sperm DNA in Pel-overproducing cultures, resist digestion with DNase I even after extended digestion [101]. eDNA returned DNase sensitive only after re-solubilizing the aggregates by sonication or increasing the pH to disrupt ionic interactions between the two polymers. This protection provided by the Pel-eDNA interaction from the disruption by nucleases has significant implications for future antibiofilm strategies. Moreover, the authors found that the Pel-eDNA interaction was also associated to an increased tolerance to cationic aminoglycoside antibiotics such as tobramycin but not to the neutral ciprofloxacin antibiotic, pinpointing a multifunctional role. Psl was not found to exhibit any of these two activities.

For many species such as *Bacillus subtilis*, the molecular structure of the exopolysaccharides found in the biofilm extracellular matrix are still unknown. Peng et al. [102] have recently reported that the expression of the main exopolysaccharide in *B. subtilis*, SBE1, may be associated to the expression of the *epsG* gene and eDNA was found to colocalize with such exopolysaccharide in *B. subtilis* SBE1 pellicles, suggesting a potential physical interaction between these two components.

Table 1 reports the polysaccharides that have been demonstrated to interact with eDNA in the biofilm architecture.

## 5. Interaction of eDNA with Biofilm Proteins

Investigations on *S. aureus* strains that produce *ica*-independent, proteinase K-sensitive biofilms have shown as treatments with proteinase K or DNase I (but not RNase) disrupt cells clumping [86]. Conversely, the addition of exogenous DNA (either autologous or heterologous) can restore cells clumping after DNase I treatment, but not in proteinase K pretreated biofilms. These observations highlight as proteins and eDNA are two interrelated main players in bacterial aggregation and biofilm stabilization. Nowadays, several proteins have emerged to interact with eDNA and contribute to the biofilm skeletal framework. Payne and Boles [103] have earlier reviewed some of the proteins that take part to the biofilm architecture and interact with eDNA. Among the different categories of proteins that have been found to express eDNA-binding activity there are: secreted proteins (e.g., the exotoxin β-toxin); proteins and lipoproteins expressed on the bacterial surface (e.g., *S. aureus* SaeP and the immunodominant surface antigen B, IsaB); nucleoid proteins and other cytoplasmic moonlighting proteins (e.g., DNABII family proteins); and amyloid/amyloidogenic proteins (e.g., phenol-soluble modulins, PSMs). Table 2 summarizes the main proteins that have been described to interact with eDNA playing a potential role in the organization of biofilm architecture.

As seen for the electrostatic net model involving polycationic exopolysaccharides and eDNA, Foulston et al. [104] suggested that eDNA might serve as an electrostatic net and link protein-coated cells in the biofilm. The authors reported as in *S. aureus* an important contribution to biofilm formation derives from the repurposing of moonlighting proteins derived from cytolysis processes. Once released in the outer space, cytoplasmic proteins would reversibly associate with the cell surface in a manner that depends on local pH. Under conditions of oxygen limitations, a drop in pH is associated to the release in the extracellular space of fermentation products, these including, among others: formate, lactate, and acetate [105]. At pH 4.5 to 5, surface-associated, cytoplasmic proteins are likely to carry a net positive charge and interact with both eDNA and with negatively charged cell surfaces characterized by the presence of polyanionic teichoic acids.

The identification of eDNA-binding proteins in the biofilms of the different bacterial species has only recently started to be unveiled. Increasing evidence has enlightened as different members of the DNABII protein family develop important interactions with eDNA and contribute to organize and stabilize the architecture of the biofilm matrix. Histone-like proteins of about 10 kDa (alternatively known as HLPs), DNABII form dimers that bind dsDNA. Initially known for their intracellular role, DNABII have progressively emerged to take part to the composition of extracellular matrix, where they critically contribute to the integrity of the biofilms containing eDNA [106,107]. Bacterial DNABII include the integration host factor (IHF) protein and the histone-like nucleoid-associated protein HU [108]. Different in vitro and in vivo studies clearly indicate a general involvement of the homologs of IHF (found in α- and γ-proteobacteria such as *Burkholderia cenocepacia* and *H. influenzae*) and of HU (broadly expressed by eubacteria), even in the biofilm architecture of relevant pathogens such as *E. coli*, *S. aureus* and *P. aeruginosa* [109,110,111]. Similarly, both IHF and HU bind dsDNA, exhibiting special affinity to pre-bent DNA. However, HU binding is not driven by specific DNA sequences, in this differing from IHF that has specific affinity for strands with the consensus WATCAANNNNTTR (W = A/T, R = A/G; N = any nucleotide; R = purine) [112]. Apart from binding of pre-bent dsDNA, HU exhibits a peculiarity with respect to many other DNA-binding proteins: an even greater affinity for cruciform branched nucleic acid structures known as Holliday junctions (HJs), up to 1000-fold higher than the affinity for double-stranded DNA or single-stranded oligonucleotides with no secondary structure [113,114]. Single-strand cross-over intermediates of homologous recombination, HJs are known to form in bacterial as well as in eukaryotic cells.

A series of interesting investigations conducted on in vivo monomicrobial and polymicrobial infections has documented that, in in vivo biofilms, eDNA is in a lattice structure [73,109,115,116,117]. Using immunohistochemical techniques, Devaraj et al. [118] were able to detect a diffuse presence of HJs in the complex lattice-like eDNA structure of biofilms formed in nontypeable *Haemophilus influenzae* (NTHI) infected middle ears of chinchillas. Similar observations were reported when analyzing the sputum of cystic fibrosis patients, where the biofilm was formed by multiple mixed bacterial species. DNABII proteins have been found to critically contribute to stabilization of the lattice structure of eDNA by binding to HJs. Indeed, HJ DNA-binding protein RuvA, normally implicated in the resolution of homologous recombination events in eubacteria, was found to functionally complement DNABII proteins within the extracellular matrix and stabilize the biofilms formed in vitro by *E. coli*, NTHI, and *S. epidermidis*. Conversely, sequestration of the DNABII proteins or competition for the HJs by HJ resolvases resulted in biofilm disruption.

Concerning the origin of eDNA-binding proteins in the extracellular space, DNABII proteins can be released in the outer space by bacterial cytolysis, but also through alternative routes as in the case of NTHI, where it occurs via inner membrane pore T4SS-like complex and ComE [117].

Apart from IHF and HU, there are several other nucleoid associated proteins (NAPs) that could potentially act as moonlighting proteins. Devaraj et al. [118] investigated if, in NTHI, NAPs other than DNABII such as H-NS, CbpA, HfQ and Dps could be found extracellularly and play an important role in the biofilm. Although NAPs other than DNABII were detected in the extracellular matrix, only DNABII were found to play a major role in maintaining the structural integrity of the biofilm. Interestingly, visualizing the biofilm with immunostaining techniques, IHF and HU were found distributed at distinct locations and did not show overlapping [118]. The critical importance of DNABII proteins had earlier been abundantly documented by a series of studies by the same group of authors, proving as the use of DNABII protein specific antibodies is capable to disrupt in vitro preformed biofilms of pathogens such as NTHI, *B. cenocepacia*, *Streptococcus gordonii* and *Porphyromonas gingivalis* and result in the dismantling and eradication of the biofilm in vivo [106,115,119,120].

Lipoproteins represent another important category of proteins capable to directly interact with eDNA and participate to the biofilm architecture as eDNA anchoring points on the cell surface. The recent work of Kavanaugh et al. [85] showed as specific membrane-attached lipoproteins can interact with the eDNA in the biofilm matrix and promote *S. aureus* biofilm formation. These authors adopted an innovative approach combining Southwestern blotting and mass spectrometry for screening the bacterial proteins capable of eDNA binding. While confirming the role of some proteins with known eDNA-binding activity (e.g., IsaB, Atl, Eap and PSMs), they also identified membrane-associated proteins and membrane-anchored lipoproteins previously unknown to bind DNA. The overexpression of identified eDNA-binding proteins was found to result in an increased retention of surface eDNA and, thus, an enhanced biofilm biomass. This finding is consistent with the hypothesis that the membrane-bound lipoproteins can function as anchor points and link bacterial cells together through noncovalent bonds with eDNA. Interestingly, one of the newly identified eDNA-binding lipoproteins by Kavanaugh and colleagues [85] was SaeP, which was previously known as an auxiliary membrane protein modulating, in concert with protein SaeQ, the activity of the SaeRS two-component system (SaeRS TCS). SaeRS TCS regulates the expression of several virulence factors (e.g., hemolysins, leukocidins, superantigens, surface proteins, and so on) and, among them, of the staphylococcal nuclease (Nuc), which has been implicated in the disruption of the biofilm during the exodus phase [121]. Therefore, SaeP could potentially express its biofilm-enhancing activity through two distinct mechanisms, respectively: (1) the inhibition of Nuc production through a modulating activity on SaeRS TCS and (2) its favorable interactions with the eDNA, strengthening the biofilm electrostatic net.

eDNA was also found to interact with and promote the polymerization of amyloidogenic peptides into self-assembled fibrillar structures. Amyloidogenic peptides represent a further category of proteins that are expressed in the biofilms of several bacterial species. For *E. coli*, a key amyloid component that binds eDNA promoting curli amyloids assembly is CsgA. Yan et al. (2020) [122] have reported some insights on the biogenesis of curli amyloids by the cooperations of CsgA with six distinct peptides. After recognition by CsgG, CsgA would be secreted through the CsgFG secretion channel into the extracellular space, where it would be eventually nucleated by CsgB and forms fibrils. In addition to *E. coli*, curli is known to be expressed also in *Salmonella enterica* serovar *Typhimurium* and in other species of the *Enterobacteriaceae* family, where it has been found to form curli-DNA complexes that contribute to the biofilm structure. Under in vivo conditions amyloid-DNA composites of bacterial biofilms have been observed and found capable to stimulate autoimmunity diseases through the generation of anti-dsDNA antibodies [55,123]. DNA was found to accelerate the polymerization of curli fibers [123]. Thus, eDNA would not only bind to curli forming complexes, but also promote the formation of stable amyloid fibrils acting on the polymerization rate. Both these actions contribute to an increased structural stability of the biofilms.

Phenol-soluble modulins (PSM) represent another group of bacterial amyloidogenic proteins capable to interact with eDNA in the extracellular space. Small surfactant-like amphipathic peptides, PSMs are multifunctional proteins that are known to act as cytotoxic virulence factors protecting bacteria from the host immune response, but also intervene in the process of staphylococcal biofilm maturation. In particular, they have been found to act as surfactant molecules and lead to biofilm disassembly when in monomeric form, but also contribute to the integrity of the biofilm architecture when self-assembled into amyloid-like fibers exhibiting amyloid-like properties [124]. Marinelli et al. [125] reported that, in *S. aureus*, α-PSM1 and α-PSM4 peptides are main amyloidogenic proteins involved in the α-PSMs fibrillogenesis. At a low concentration PSMs monomers alone do not readily polymerize. Nonetheless, positively charged PSMs such as α-PSM1 would interact with polyanionic eDNA, thus raising the local peptide concentration and triggering polymerization [126]. The formation of the DNA/PSM complex was also found to reduce the cytolytic activity of α-PSM10 [126], suggesting that the presence of eDNA would modulate at the same time biofilm stability and leukocidal action toward host cell-mediated response. It would be interesting to investigate if other eDNA-binding substances could play a kind of regulatory function by competing with the binding of PSMs.

*S. aureus* immunodominant surface antigen B (IsaB) is a further eDNA-binding protein, which is secreted in the extracellular space, where it remains retained on the bacterial surface [127]. Elevated IsaB has been reported to inhibit host autophagic flux and promote MRSA virulence. It remains a matter of speculation if its eDNA binding activity could play a further role in anchoring nucleic acids to the cell surface [128]. In addition to IsaB, DNA-binding activity is expressed also by the β-Toxin, a neutral sphingomyelinase hemolysin, which forms covalent cross-links to itself in the presence of DNA [129]. However, even in this case, its role in the biofilm structure is still to be completely clarified.

**Table 2:** Proteins interacting with eDNA in the biofilm architecture.

Component	Category	Description	Main Bacterial Species	References
IHF	DNABII protein family	Integration host factor protein	α- and γ-proteobacteria	[106,107,108,109,110,111]
HU	DNABII protein family	Histone-like nucleoid-associated protein HU	Broadly expressed by eubacteria	[106,107,108,109,110,111]
DNABII proteins	Protein	Bind at the vertices of crossed eDNA strands and act as lynchpin-like molecules to stabilize the structure of eDNA	Nontypeable *Haemophilus influenzae* (NTHI)	[117]
SaeP	Lipoprotein	SaeP inhibits Nuc production through a modulating activity on SaeRS TCS and interacts with the eDNA, strengthening the biofilm electrostatic net.	*S. aureus*	[85]
β-toxin	Exotoxin	A neutral sphingomyelinase hemolysin, β-toxin has been speculated to form covalent cross-links to itself in the presence of DNA	*S. aureus*	[129]
IsaB	Extra-cellular protein retained on bacterial surface	*S. aureus* immunodominant surface antigen B (IsaB) has been speculated to anchor nucleic acids to the bacterial cell surface	*S. aureus*	[128]
LytC	Extra-cellular protein	Lysozyme LytC, an extra-cellular cell wall hydrolase, has been suggested to form DNA-LytC protein complexes	*S. pneumoniae*	[130]
PrgB	Surface adhesin	Its adhesin domain has been reporteed to bind and compact DNA	*Enterococcus faecalis*	[131,132]
CsgA	Amyloid/amyloidogenic proteins	Amyloid component that binds eDNA promoting curli amyloids assembly	*E. coli, Salmonella enterica* serovar *Typhimurium, Enterobacteriaceae* family	[122]
α-PSM1	Amyloid/amyloidogenic proteins	A positively charged phenol-soluble modulin (PSM), α-PSM1 can interact with polyanionic eDNA and trigger α-PSMs fibrillogenesis	*S. aureus*	[124,125,126]

Other bacterial factors described to interact with eDNA and hypothesized to play a function in stabilizing the biofilm architecture include: lysozyme LytC, a *S. pneumoniae* extra-cellular cell wall hydrolase that forms intercellular DNA-LytC protein complexes in pneumococcal biofilms [130]; *Enterococcus faecalis* PrgB, whose adhesin domain binds and compacts DNA with a histone-like DNA condensation mechanism [131,132]; *Streptococcus intermedius* histone-like DNA-binding protein (Si-HLP) [133,134]; GAPDH and enolase *S. aureus* proteins, which are positively charged at pH 5 [81,86,104]; and *Neisseria* heparin-binding antigen (NhbA) and α-peptide of IgA protease both expressed by *Neisseria meningitidis* [135].

## 6. Bacterial eDNA Binding to Human Inflammatory Cells

All microbial structural motifs are recognized by the innate immune system via the TLR family of pattern recognition receptors (PRRs) [136]. TRL9 is a pattern recognition receptor, which is predominantly located intracellularly in immune cells and recognizes unmethylated CpG motifs characteristic of bacterial DNA [44,45]. TLR9-dependent activation can be triggered not only by phagocytosis of whole *S. aureus* cells but also by that of the extracellular DNA molecules, extensively contained in the biofilm matrix. The role of TLR9 in regulating host immunity to *S. aureus* biofilm growth has been examined by Thurlow et al. [137]. Although TLR9 is pivotal for host immune responses to planktonic *S. aureus*, immune response appears different in *S. aureus* biofilm infections. Using a mouse model of catheter-associated biofilm infection, these authors demonstrated a significant reduction in cytokine/chemokine production associated with biofilm in infected tissues compared with the wound healing response elicited by sterile catheters and concluded that *S. aureus* biofilms can circumvent traditional antimicrobial effector pathways and persist in an immuno-competent host. Human inflammatory cells rapidly detect CpG sequences in bacterial eDNA as a human evolutionary response to combat the ability of bacteria to rapidly form biofilm communities. In wild type mice, intraperitoneal application of small synthetic oligodeoxynucleotides containing unmethylated CpG dinucleotides has been found to lead to both a local and systemic inflammatory response. Conversely, in TLR9-deficient mice, the inflammatory response was abolished [138]. These findings would suggest that bacterial eDNA would potentially influence the inflammatory response even systemically [138].

In immune cells, the recognition of bacterial extracellular DNA by a surface receptor has evolved as a signal to alert the immune system to the presence of bacterial invasion, mainly in infections in which bacteria grow as biofilms. This effect might be relevant in biofilm infections in which extracellular DNA could function as a potent neutrophil agonist [139].

However, whether TLR9 plays a protective or deleterious role in host defense against *P. aeruginosa* remained to be determined. TLR9 plays an essential role in activating innate immunity by recognizing CpG specific motifs present in microbial DNA [10,140]. It is however reported that TLR9 down-regulates the innate immune response against *P. aeruginosa* and that the absence of TLR9 leads to an early increase in the inflammatory response [141]. This leads to the improvement of the clearance of *P. aeruginosa* in the lungs and increased mouse survival. The apparent enhancement in the ability of TLR9-negative mice to eliminate *P. aeruginosa* seems due to a TLR9 deletion that improves alveolar macrophages killing of this bacterium by increasing the production of IL-1b and NO, two inflammatory mediators necessary for *P. aeruginosa* killing by alveolar macrophages. According to the authors, this finding may open therapeutic strategies, based on TLR9 inhibition, to control *P. aeruginosa*-induced pneumonia.

## 7. Interaction of eDNA with Other Substances/Metabolites

Apart from the binding to other EPS components such as exopolysaccharides and proteins, eDNA has been shown to interact also with substances of different nature and functions. Redox-active metabolites like phenazines have been found to bind DNA through intercalation. In *P. aeruginosa*, pyocyanin binding to e-DNA has been reported to increase EPS stability and cellular aggregation [142]. In reason of its electrostatic charge, eDNA is known to complexate with polycationic ions and substances. For instance, the interaction between DNA and Ca2+ was found thermodynamically favorable and the binding process is spontaneous and exothermic. At biologically relevant concentrations, Ca2+ was found to enhance cell aggregation via cationic bridging of DNA [143]. Moreover, through its drug binding activity, eDNA confers to the biofilm a shielding function, protecting the encased bacteria from the bactericidal action of antibiotics as well as antimicrobial peptides [42,144]. Finally, eDNA has also been found to interact with supramolecular materials such as membrane vesicles [145], which have been observed in the biofilm to associate to the nucleic acid.

## 8. Attachment to Abiotic Surfaces and Bridging Processes

In the biofilm architecture eDNA has been seen to interact with different substances and form a diffuse web-like interconnected lattice structure. This diffuse net, we have seen, can be anchored to the bacterial surfaces through externally exposed lipoproteins and other surface associated eDNA-binding proteins. Eventually, polycationic substances of polysaccharidic or proteic nature may additionally interact with other competing anionic superficial bacterial molecules such as lipoteichoic acids and interlace these molecules with eDNA. The action of all these intermolecular interactions would explicate cell aggregating activity and letting emerge a clear role for eDNA as an intercellular adhesin. The heterogeneity of environmental conditions within a biofilm, in particular local pH, dictates the electrostatic charges of EPS components, so affecting the interactivity of the biofilm architectural elements. Physicochemical mechanistic explanations for eDNA-mediated adhesion and aggregation come also from the extended Derjaguin, Landau, Verwey, Overbeek (DLVO) theory. In this perspective, eDNA-mediated bacterial adhesion and aggregation would occur through attractive Lifshitz–Van der Waals and acid–base interactions despite existing electrostatic repulsion. In the extended DLVO theory, acid–base attraction results from interactions between electron-accepting and electron-donating moieties on cells surfaces and eDNA [146,147]. Attractive short-range acid-base interactions would contribute to eDNA binding with other EPS biopolymers and other intercalating metabolites such as pyocyanin, facilitating EPS anchoring on bacterial cell surfaces.

When bacteria are suspended in a fluid, in the absence of a substrate to adhere to and form a stratified biofilm, autoaggregation (also termed flocculation or agglutination) is believed to provide bacteria with the benefits of biofilm while maintaining mobility [148]. This is the case of many chronic, recalcitrant bacterial infections such as cystic fibrosis, where bacteria can persist and elude both host immune defenses and medical antibiotic therapies forming mono- or polymicrobial biofilms in the absence of a foreign body.

In the presence of materials with slippery, non-adhesive surfaces, eDNA has been found to play a central role in the formation of “biofilm bridges”. Biofilm bridging is a newly discovered phenomenon. Bacterial microcolonies do not remain confined to restricted adhesive surface microniches on the substrate but can successfully spread across bacteria-repulsive regions and develop contact with other distant biofilm clusters. All this is accomplished by forming thin biofilm bridges, which originate as a stress response to harsh environmental conditions (e.g., limited iron ion availability). The formation of biofilm bridges has been demonstrated to occur in *P. aeruginosa*, *S. aureus* and *Stenotrophomonas maltophilia* [149]. The significant reduction in the formation of biofilm bridges when treating bacterial cultures with DNase clearly pinpoints to the involvement of eDNA in the process. Indeed, eDNA was detected not only within the bridge but also in the adjacent areas, encountering the main biofilm clusters. However, apart from its structural role, eDNA has also been found to facilitate twitching motility of bacteria and, thus promote migration and biofilm spreading. eDNA would explicate the activity on cell motility by orderly directing the traffic flow of cells to the leading edges of biofilm through a sort of contact guidance [52].

Apart from intervening as an intercellular glue, eDNA has also been found to mediate and influence bacterial adhesion on material surfaces, which represents a fundamental step in the pathogenesis of biofilm-associate infections developing in the presence of foreign bodies and implant materials. Regina et al. [150] found that eDNA promotes bacterial adhesion to abiotic surfaces characterized by a wide range of surface chemistries. However, its effects on bacterial adhesion were found not only to depend on the material surface chemistry but also on the ionic strength (I) of the surrounding fluid [150,151]. eDNA would participate both in short-range acid-base interactions and long-range electrostatic and Lifshitz-van der Waals interactions. On hydrophilic surfaces, eDNA-stimulated *Staphylococcus xylosus* bacterial adhesion was only evident at low and intermediate I, while no effect was seen on glass surfaces and carboxyl-functionalized surfaces at high I of the surrounding liquid [150]. Conversely, eDNA was found to create favorable conditions for bacterial adhesion to neutrally charged hydrophobic surfaces, independently from I [150]. DNA is an amphipathic molecule consisting of a hydrophilic backbone and hydrophobic nitrogenous bases, which are potentially implicated in hydrophobic interactions with bacterial surfaces.

**Figure 2:**
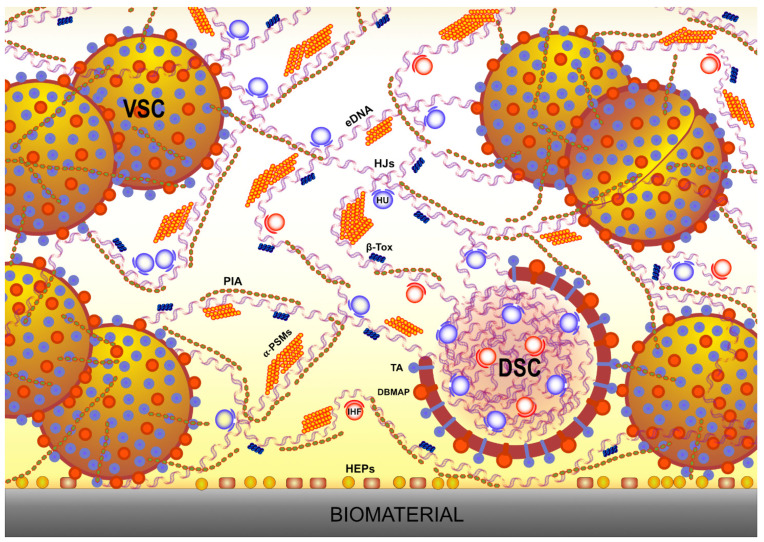
This scheme attempts to illustrate the various and complex molecular interactions taking place within the matrix of staphylococcal biofilms. Water-soluble molecules are retained by their mutual stabilizing interactions. The attention is focused on the variety of molecular species emerged to be involved and the real size ratio among different categories of molecules is not fully respected. During biofilm maturation, eDNA becomes progressively protected by the complexation with the other polymers and resistant to DNase digestion. Legend: VSC, viable staphylococcal cell; DSC, dead staphylococcal cell; HJs, eDNA Holliday junctions; PIA, polysaccharide intercellular adhesin; HU, histone-like nucleoid-associated protein HU; IHF, integration host factor; SaeP, *S. aureus* Saep lipoprotein; TA, (negatively charged) teichoic acids; β-Tox, *S. aureus* β-Toxin; α-PSMs, amyloidogenic α-Phenol-soluble modulins; DBMAP, DNA-binding membrane-associated proteins and membrane-anchored lipoproteins such as *S. aureus* SaeP and IsaB; HEPs, host extracellular-matrix proteins.

Figure 2 graphically summarizes some principal interactions emerged to occur in the biofilm of *S. aureus*, an exemplary, largely investigated staphylococcal species.

## 9. Conclusions

Biofilm formation is being universally recognized as a principal virulence mechanism enabling bacteria to elude the host immune response and circumvent conventional antibiotic treatments. Thus, bacterial growth in the biofilm state has progressively become the primary target of an increasing number of strategies aimed at preventing and eradicating difficult to treat clinical infections. However, as also pointed out by Flemming et al. [88], the concept of biofilm has been and is constantly evolving and human categories are often difficult to adapt for interpreting complex natural phenomena still incompletely understood. Surprisingly, for many bacterial species there is still very limited knowledge available on the biofilm matrix composition and the structural and functional role of each single molecule that may be expressed during different phases of biofilm maturation. A high degree of biodiversity is overall observed, with biofilms markedly varying in composition depending on bacterial species, strain types, stage of maturation and environmental conditions. Nonetheless, eDNA appears to be widespread and ubiquitously participate to the biofilm matrix composition of most bacterial and fungal species. This versatile molecule exhibits physico-chemical properties that are ideal to cover structural and functional roles required to microbial cells to strive in different environments, including the hostile interstices of the human body. Based on convergent recent findings, eDNA consistently emerges as a main structural element of a self-assembling architecture, which coordinates and bind a broad range of compounds secreted by bacteria, present in the environment, or provided by the host. Its chemistry enables a prompt interaction, often based on electrostatic charges, with molecules of various nature, promotes the formation of stabilizing eDNA networks and the spontaneous assembly of supramolecular structures that are fundamental for bacterial intercellular aggregation, bacterial anchorage to the substrate and formation of a functional and definite biofilm architecture. In view of its wide distribution across biofilms formed by main pathogenic species, eDNA probably represents the best target for broadly addressing anti-biofilm strategies. An important point is however the decreased vulnerability to DNase degradation acquired by biofilms during their maturation. It was initially thought that eDNA cornerstone function during the initial morphogenesis of biofilm matrix was finally supplanted/superseded by that of other EPS constituents. However, the protective complexation/interaction of other EPS components, in first place amyloidogenic and DNABII proteins, with eDNA has been found a critical point of vulnerability and an Achilles’ heel of biofilm structural integrity. These latest advancements in the knowledge of the biofilm matrix architecture are already inspiring new powerful therapeutic approaches to efficaciously treat and prevent biofilm-based infections, particularly the dreaded and multifaceted infections associated with orthopedic implants.

## Figures and Tables

**Table 1:** Polysaccharides interacting with eDNA in the biofilm architecture.

Component	Category	Description	Main Bacterial Species	References
PIA, PNAG	Exopolysaccharide	poly-β-(1,6)-*N*-acetylglucosamine. In *S. aureus* and *S. epidermidis* PIA represents a main exopolysaccharide biofilm component	*S. aureus, S. epidermidis*	[17,25,82,83,84]
Pel polysaccharide	Exopolysaccharide	Polycationic, partially de-*N*-acetylated polysaccharide consisting of *N*-acetylglucosamine and *N*-acetylgalactosamine	*P. aeruginosa*	[92,93,94,95,96,97,100]
Psl polysaccharide	Exopolysaccharide	Neutral mannose-rich polysaccharide, whose direct interaction with eDNA is still debated	*P. aeruginosa*	[92,93,94,95,96,98]

## Data Availability

Not applicable.
